# Combined closed surgery with ADSCs, photons and PRP. The science and practice behind this new single stage method of rejuvenation

**DOI:** 10.1186/1471-2334-13-S1-P57

**Published:** 2013-12-16

**Authors:** Dana Mihaela Jianu, Maria Filipescu, Ștefan Adrian Jianu, Ioan Florescu, Andreea Nita, Oltjon Cobani

**Affiliations:** 1 Proestetica Medical Center, Bucharest, Romania; 2 Carol Davila University of Medicine and Pharmacy, Bucharest, Romania

## Background

“From filling to regeneration” - a new horizon is opened: regenerative surgery. Cellular therapies (surgery) tend to replace the old scalpel or at least to offer alternatives. The potential of adipose-derived stem cells (ADSCs) seems to be optimized by energy (photons) and platelet-rich plasma (PRP).

Subtle, but very powerful tools coming from science to serve in softness our daily surgical practice aiming attractiveness and rejuvenation.

## Method

A relevant number of patients (200) are demonstrating that head and neck rejuvenation and beautification can be obtained satisfactory in the most critical points: 1. Perioculo-malar area; 2. Mandibular line; 3. Mandible neck angle; 4. Skin texture; if using simultaneously a combination of regenerative therapies: A. facial fat transplant, B. 2 types of LASERS (optical fiber DIODE and resurfacing CO2 Fractional), C. PRP.

## Results

Beside the well-known repercussion of each A, B, C the empowering cumulative effect is to be highlighted: fat transplant is bringing not only volume but ADSCs. They are stimulated by photonic energy (delivered by LASERS), the healing of burns is accelerated after PRP due to their growth factors which increase the quality of regeneration. These cellular therapies lead to a younger look with fast recovery, no incisions, less complications. Histology and series of results are shown.

## Conclusion

The empowered effects of ADSCs, photonic energy and PRP can be brought together in a reproductible regenerative tissular algorithm. In authors’ opinion, although improvable as technique, the concept within this complex method, relevant for regenerative surgery will have a place in the future.

